# The *KRAS*-Variant Is Associated with Risk of Developing Double Primary Breast and Ovarian Cancer

**DOI:** 10.1371/journal.pone.0037891

**Published:** 2012-05-25

**Authors:** Robert Pilarski, Divya A. Patel, Jeffrey Weitzel, Terri McVeigh, Jemima J. Dorairaj, Helen M. Heneghan, Nicola Miller, Joanne B. Weidhaas, Michael J. Kerin, Megan McKenna, Xifeng Wu, Michelle Hildebrandt, Daniel Zelterman, Sharon Sand, Lee P. Shulman

**Affiliations:** 1 The Ohio State University Comprehensive Cancer Center and Department of Internal Medicine, Columbus, Ohio, United States of America; 2 Department of Obstetrics, Gynecology and Reproductive Sciences, Yale University, New Haven, Connecticut, United States of America; 3 Division of Clinical Cancer Genetics, City of Hope, Duarte, California, United States of America; 4 Discipline of Surgery, National University of Ireland, Galway, Ireland; 5 Department of Therapeutic Radiology, Yale University, New Haven, Connecticut, United States of America; 6 Cancer Center of Santa Barbara, Santa Barbara, California, United States of America; 7 Department of Epidemiology, The University of Texas MD Anderson Cancer Center, Houston, Texas, United States of America; 8 Department of Epidemiology and Public Health, Yale University, New Haven, Connecticut, United States of America; 9 Division of Clinical Genetics, Feinberg School of Medicine, Northwestern University, Chicago, Illinois, United States of America; Health Canada, Canada

## Abstract

**Purpose:**

A germline microRNA binding site-disrupting variant, the *KRAS-*variant (*rs61764370*), is associated with an increased risk of developing several cancers. Because this variant is most strongly associated with ovarian cancer risk in patients from hereditary breast and ovarian families (HBOC), and with the risk of premenopausal triple negative breast cancer, we evaluated the association of the *KRAS-*variant with women with personal histories of both breast and ovarian cancer, referred to as double primary patients.

**Experimental Design:**

Germline DNA from double primary patients was tested for the *KRAS*-variant (*n = *232). Confirmation of pathologic diagnoses, age of diagnoses, interval between ovarian cancer diagnosis and sample collection, additional cancer diagnoses, and family history were obtained when available. All patients were tested for deleterious *BRCA* mutations.

**Results:**

The *KRAS-*variant was significantly enriched in uninformative (*BRCA* negative) double primary patients, being found in 39% of patients accrued within two years of their ovarian cancer diagnosis. Furthermore, the *KRAS-*variant was found in 35% of uninformative double primary patients diagnosed with ovarian cancer post-menopausally, and was significantly associated with uninformative double primary patients with a positive family history. The *KRAS-*variant was also significantly enriched in uninformative patients who developed more then two primary cancers, being found in 48% of women with two breast primaries plus ovarian cancer or with triple primary cancers.

**Conclusions:**

These findings further validate the importance of the *KRAS-*variant in breast and ovarian cancer risk, and support the association of this variant as a genetic marker for HBOC families previously considered uninformative.

## Introduction

Hereditary breast and ovarian cancer (HBOC) syndrome is an inherited cancer-susceptibility syndrome marked by an increased risk of developing both ovarian cancer and breast cancer [1]. Families generally considered as having HBOC syndrome are those with multiple family members that have one of these cancers, especially at young ages, or an individual with a cancer in both organs, a “double primary” patient. While this is a relatively rare presentation, a substantial number of women develop both breast and ovarian primaries over their lifetime. While *BRCA1* and *BRCA2* are strongly associated with HBOC syndrome [2], a large number of HBOC families and women with double primary cancer do not have detectable genetic mutations (herein referred to as “uninformative” patients).

The chances of identifying a mutation causative for HBOC increase when testing individuals diagnosed with double breast/ovarian primaries [3]–[5]. However, a recent report suggests that the rates of *BRCA* mutations are not higher in a patient with a double primary without a family history than that for isolated first degree relative pairs with single primaries (14% versus 17% with mutations, respectively) [4]. This supports the importance of family history even in patients with double primary cancers. Although *BRCA* mutations were found in 49% of double primary patients in this recent analysis, it should be noted that this indicates that over half of double primary patients do not have a known genetic cause for their disease. This is consistent with other reports of these patients [3], [5].

Many women diagnosed with premenopausal breast cancer undergo testing for *BRCA* mutations, and many do this to gain information on their future ovarian cancer risk [3], [6]. For these women this may be the most important role of genetic testing, as positive testing could allow prevention or early detection of ovarian cancer [7]. Furthermore, current evidence suggests that women with breast cancer who are negative for *BRCA* mutations are not at an increased risk of developing ovarian cancer in the absence of a significant family history of ovarian cancer [8].

Previously, there have not been additional genetic markers associated with risk of disease in both the breast and the ovary besides *BRCA1* and *BRCA2*. However, a functional germline variant in the 3′UTR of the *KRAS* oncogene (*rs61764370*) has been recently identified and reported to be associated with increased risk of both invasive epithelial ovarian cancer [9] and breast cancer [10] in clinically well-annotated cohorts. The association of the *KRAS*-variant with ovarian cancer was most significant for uninformative women from HBOC families, and the association with breast cancer was significant for premenopausal women with triple negative breast cancer, also often indicative of an HBOC family.

The goal of this study was to determine the association of the *KRAS*-variant with women with double primary breast and ovarian cancer, to further validate the association of this variant with HBOC families. Findings here support the importance of the *KRAS-*variant in uninformative HBOC families as well as in predicting the risk of multiple primary cancers in women.

## Methods

### Ethics Statement

All patients in this study were consented and enrolled on institution protocols for DNA collection by written consent. Institution review boards and ethic committees that approved this study were City of Hope, Memorial Sloan Kettering Cancer Center, The University of Texas MD Anderson Cancer Center, Yale University, Ohio State University, Northwestern University, Cancer Center of Santa Barbara and National University of Ireland.

**Table 1:** The *KRAS-*variant is significantly associated with uninformative breast and ovarian cancer patients.

	*BRCA1 (n = 75)*	*BRCA2 (n = 33)*	*Uninformative (n = 92)*
Prevalence	16.0%	18.2%	27.2% (p<0.001)

### Patients

Patients from eight separate institutions (City of Hope, Memorial Sloan Kettering Cancer Center, The University of Texas MD Anderson Cancer Center, Yale University, Ohio State University, Northwestern University, Cancer Center of Santa Barbara and National University of Ireland) were recruited under standard individual institution approved IRB protocols for DNA sample collection (total *n = *232). Double primary patients from Yale University were prospectively collected for this study. Each patient had pathologically documented double primary cancer - breast cancer and invasive epithelial ovarian cancer. For a patient’s breast cancer diagnosis, ductal carcinoma in situ (DCIS), invasive lobular or invasive ductal cancers were eligible for study inclusion. For a patient’s ovarian cancer diagnosis, epithelial ovarian cancer, fallopian tube cancer or primary peritoneal cancers were eligible for study inclusion. All patients had clinical testing for *BRCA* mutations by sequencing, and uninformative patients had no sequencing variants. Deletion/duplication testing was not done in most subjects.

In the analysis, samples from 75 patients with pathogenic *BRCA1* mutations, 33 patients with pathogenic *BRCA2* mutations, and 124 uninformative (i.e., negative for *BRCA* mutations) patients were analyzed for the *KRAS*-variant. Patient demographics including ethnicity, age at breast and ovarian cancer diagnosis, additional cancer diagnoses, time between ovarian cancer diagnosis and sample collection, and family history were recorded at each institution for most patients when available (all *BRCA1* and *BRCA2,* 92 uninformative, *n* = 200). An additional cohort of uninformative patients with only known diagnosis and detailed family history (from Memorial Sloan Kettering Cancer Center) were included to better study the impact of family history on *KRAS*-variant status in women with double primary cancers (*n = *32), for the total cohort size of 232. Postmenopausal status was estimated as age 52 years or older for all patients.

### Assay

Germline DNA from each patient was isolated from blood or saliva and stored using standard protocols. Germline DNA was assayed for the *KRAS-*variant using a Taqman custom designed assay (ABI, CA) with relevant positive and negative cell line DNA controls. Samples were analyzed at the individual parent institution (*n* = 95), at Yale University in a blinded fashion (*n* = 64), or at Mira Dx, Inc. (New Haven, CT), a Clinical Laboratory Improvement Amendment (CLIA) certified laboratory (*n* = 73).

### Statistical Methods

The prevalence of the *KRAS-*variant was examined in relation to ethnicity, *BRCA* mutation status, time between ovarian cancer diagnosis and recruitment, age of ovarian cancer onset, family history and multiple primary cancers. Small frequency distributions were compared using Fisher’s exact test and comparisons with population rates (n>6800) using a binomial model. Logistic regression models were used to examine the association between subject age and the *KRAS*-variant. P-values less than.05 were considered statistically significant. All the analyses were performed using SAS software (Version 9.2) or in R (Version 2.12).

**Table 2:** The *KRAS-*variant is significantly more likely to be found in women tested within two years of their ovarian cancer diagnosis.

	Overall (*n = 82*)	<2 years from ovarian cancer diagnosis (*n = 52*)	>2 years from ovarian cancer diagnosis (*n = 30*)
Prevalence	30.5%	38.5%	16.7%

## Results

### Prevalence of the *KRAS*-variant in Double Primary Patients by Ethnicity

Overall, the *KRAS*-variant was found in 21.0% of the entire cohort of double primary breast and ovarian cancer patients with full clinical information (*n* = 42/200). This is significantly higher than the population prevalence of ∼15% observed in non-cancerous Caucasian control populations (p = 0.01 binomial test)[9]–[13]. Because the baseline prevalence of the *KRAS*-variant varies across ethnic populations [11], and is highest in Caucasian non-Hispanic populations, we examined the prevalence of the *KRAS*-variant in Caucasian non-Hispanic double primary patients only, and found the prevalence of the *KRAS*-variant was slightly higher in these women compared to the overall cohort (38/163 = 23.3%, p = 0.002, binomial). The difference in prevalence of the *KRAS-*variant between Caucasian non-Hispanic, and non-Caucasian or Hispanic women with double primary cancer was not significant, however (p = 0.6), indicating that the *KRAS*-variant is significantly associated with double primary cancer for women of all ethnicities. Therefore all double primary patients, regardless of ethnicity, were included in the additional analyses.

### The Association of the *KRAS*-variant with *BRCA* Status

We evaluated the prevalence of the *KRAS*-variant in double primary patients with full clinical information based on *BRCA* mutation status: pathogenic *BRCA1* mutations (*n = *75), pathogenic *BRCA2* mutations (*n = *33), or *BRCA*-negative (uninformative) (*n* = 92). The *KRAS*-variant was not statistically significantly elevated in women with pathogenic *BRCA1* mutations (*n* = 12/75, 16.0%), or in women with pathogenic *BRCA2* mutations (*n* = 6/33, 18.2%) compared to population prevalence. In contrast however, the prevalence of the *KRAS-*variant was significantly enriched in uninformative double primary cancer patients compared to population prevalence (25/92, 27.2%, p<0.001, binomial) (**Table 1**).

**Table 3:** The *KRAS-*variant is significantly associated with developing ovarian cancer post-menopausally compared to pre-menopausally.

	Women with post-menopausal ovariancancer (*n = 63*)	Women with pre-menopausal ovariancancer (*n = 24*)
Prevalence	34.9%	12.5%

### Impact of Interval Between Ovarian Cancer Diagnosis and Patient Recruitment on *KRAS*-variant Prevalence in Uninformative Patients

Because the *KRAS*-variant predicts poor ovarian cancer specific survival in uninformative patients [14], we investigated the association of the prevalence of the *KRAS*-variant and time between ovarian cancer diagnosis and study recruitment for uninformative patients with available information (*n* = 82). First, we found that the interval between ovarian cancer diagnosis and sample collection was significantly different across the recruitment centers, likely due to center referral patterns (p<0.001). The overall prevalence of the *KRAS*-variant was 30.5% (*n = *25/82) in uninformative patients with available information on interval between diagnosis and recruitment. The prevalence of the *KRAS-*variant was 38.5% (*n* = 20/52) in patients recruited within two years of their ovarian cancer diagnosis, which was significantly higher than the prevalence in patients recruited more than 2 years after their ovarian cancer diagnosis (16.7%, *n = *5/30, p<0.048 by Exact test) (**Table 2**).

### Timing of Ovarian Cancer Development in *KRAS*-variant Uninformative Patients

The majority of uninformative women in these studies developed breast cancer before their ovarian cancer (74.7% of all uninformative patients [*n = *65/87]). This was slightly less common in *KRAS-*variant-positive uninformative patients (64%, *n = *16/25) compared to *KRAS-*variant-negative uninformative patients (79.0%, *n = *49/62), but this difference was not significant. Because prior reports have found that the *KRAS*-variant is rarely associated with premenopausal ovarian cancer (less then 52 years of age) [9], [14], we next evaluated the association of the *KRAS*-variant with age of ovarian cancer development in uninformative double primary patients. We found that 88.0% of *KRAS*-variant-positive uninformative patients developed ovarian cancer postmenopausally (*n = *22/25), compared to only 66.1% of *KRAS*-variant-negative uninformative patients (*n = *41/62), however this difference did not reach statistical significant (p = 0.062). We additionally found a significant association of the *KRAS*-variant with age of ovarian cancer diagnosis, with 34.9% of women diagnosed with ovarian cancer postmenopausally having the *KRAS-*variant (*n = *22/63), compared to only 12.5% of women diagnosed with ovarian cancer premenopausally (*n = *3/24). This association with older age of ovarian cancer onset in *KRAS*-variant-positive uninformative patients was significant by logistic regression analysis (p<0.007) (**Table 3**).

**Table 4:** The *KRAS*-variant is significantly associated with the risk of developing additional cancers beyond breast and ovarian cancer.

	Breast and ovarian cancer(*n = 145*)	Two breasts and ovarian(*n = 22*)	Triple primary cancer(*n = 16*)
Prevalence overall	20.0%	22.7%	43.8%
Prevalence in uninformative	22.7% (15/66)	57.1% (4/7)	42.9% (6/14)

### Association of the *KRAS*-variant with Family History in Uninformative Patients

As the association of double primary cancers and known genetic mutations has been found to be enriched in the presence of a positive family history of related cancers, we evaluated the association of the prevalence of the *KRAS*-variant with family history in uninformative patients. We added an additional cohort of 32 uninformative double primary patients with a known family history to the 44 uninformative patients with known family history from our fully annotated cohort. In these 76 women with double primary cancers, 24 had a positive family history and 52 had a negative family history for breast and/or ovarian cancer in first and/or second-degree relatives. The *KRAS*-variant was found in 29.2% (7/24) of women with a positive family history, which is a prevalence significantly higher than expected in the general population (p<0.02). In contrast the *KRAS-*variant was not significantly elevated in uninformative double primary patients with a negative family history compared to the general population prevalence, being found in 15.3% (8/52) of this population. The difference between the prevalence of the *KRAS-*variant in women with a positive versus negative family history was not significant (p = 0.13).

### Association of the *KRAS*-variant with Multiple Cancers in All Patients

Because the *KRAS*-variant has been found to be associated with an increased risk for other cancers besides breast and ovarian cancer [11], [15] we tested the hypothesis that the *KRAS*-variant would predict for an increased risk of developing additional cancers in this double primary cohort, regardless of *BRCA* mutation status. For 183 of the patients in our study where this information was available, 79.2% (*n* = 145) had reported just the two cancers (breast and ovarian), 12.0% (*n* = 22) had two separate primary breast cancers and also ovarian cancer, and 8.7% (*n* = 16) had cancer in an additional organ outside of the breast and ovary (triple primary).

The *KRAS-*variant was found in 20.0% (*n = *29/145) of double primary patients overall; 19.3% (11/57) of *BRCA1* patients, 13.6% (3/22) of *BRCA2* patients and 22.7% (15/66) of uninformative patients. The *KRAS-*variant was found in 22.7% (*n = *5/22) of patients with two separate primary breast cancers and ovarian cancer; 0% (0/12) of *BRCA1* patients, 33.3% (1/3) of *BRCA2* patients and 57.1% (4/7) of uninformative patients. Finally, the *KRAS*-variant was found in 43.8% (*n = *7/16) of women with triple primaries; 0% (0/1) of *BRCA1* patients, 100% (1/1) of *BRCA2* patients, and 42.9% (6/14) of uninformative patients. The *KRAS-*variant predicts a significant increased risk of developing a third independent cancer in all double primary patients (p<0.01), which was largely due to increased risk for uninformative patients (p<0.005) and also possibly *BRCA2* patients (p<0.05). The *KRAS-*variant also predicts a significantly increased risk of developing more then two primary cancers in uninformative double primary patients, being found in 47.6% (10/21) of uninformative patients with more then two primary cancers compared to 22.7% (15/66) of uninformative patients with just two primary cancers (p = 0.05) (**Table 4**).

**Table 5:** Prevalence of the *KRAS-*variant in uninformative patients.

	YES	NO	p-value
Accrued within 2 years ofovarian cancer diagnosis	38.5%	16.7%	0.048
Developed ovarian cancerpost-menopausally	34.9%	12.5%	0.007
Developed more than twoprimary cancers	47.6%	22.7%	0.05

## Discussion

Here we show that the *KRAS*-variant, a functional germline miRNA-binding disrupting mutation that has previously been shown to be associated with ovarian cancer, especially in HBOC families [9], as well as with premenopausal triple negative breast cancer [10], is also significantly enriched in women who develop both breast and ovarian cancer with uninformative *BRCA* sequencing results (**Table 5**). The *KRAS-*variant was most enriched in women who were tested within two years of their ovarian cancer diagnosis, likely reflecting the increased risk of interim death of *KRAS-*variant positive ovarian cancer patients with longer accrual times [9]. In addition, the *KRAS-*variant was significantly associated with *BRCA*-uninformative patients who developed ovarian cancer post-menopausally (as estimated by age >52 years), and with *BRCA*-uninformative patients with a positive family history of breast or ovarian cancer. Finally, the *KRAS*-variant was significantly associated with an increased risk of developing a third, independent cancer in addition to breast and ovarian cancer, being found in 43.8% of patients with triple primary cancers, most of whom had uninformative *BRCA* testing. It is possible that a small proportion of cases considered *BRCA*-uninformative may harbor a large rearrangement mutation, known to account for about 10% of deleterious *BRCA1* mutations [16], [17] given the lack of screening in many cases. However, this would not have altered the significance of the primary observations in this report. These findings further confirm that the *KRAS-*variant is indeed a bona fide marker for uninformative HBOC families, and also highlights some similarities as well as some differences between *KRAS-*variant patients and *BRCA* mutant patients.

Because the great majority of *KRAS-*variant double primary patients in this study developed breast cancer before their ovarian cancer, it appears that there could have been an opportunity for ovarian cancer prevention through chemoprevention (oral contraceptives) and/or prophylactic oophorectomy for these women. In addition, the association of the *KRAS-*variant primarily with postmenopausal ovarian cancer suggests that oophorectomy might be reasonable delayed in these patients compared to recommendations for women with *BRCA* mutations, where oophorectomy is recommended at 35 or upon completion of childbearing. Currently, women with premenopausal breast cancer who are uninformative for *BRCA* mutations without a family history of ovarian cancer are told that they have no increased risk of ovarian cancer, based on a study of hereditary breast cancer families [7]. Our findings here indicate that women with the *KRAS-*variant are also at an increased risk of subsequently developing ovarian cancer, and should be managed accordingly.

The finding that the prevalence of the *KRAS*-variant is significantly higher in women tested within two years of ovarian cancer diagnosis likely reflects the fact that these patients have worse ovarian cancer specific survival and a higher risk for interim death over time [14]. In addition, the significant association of the *KRAS-*variant with early onset triple negative breast cancer [10], the most deadly form of breast cancer, would also have likely diluted the prevalence of the *KRAS-*variant in these cohorts, as these women would be more likely to die of their breast cancer before development of ovarian cancer. Regardless, the prevalence of the *KRAS-*variant remained significantly enriched in these patients even when studying the group as a whole. Importantly though these findings highlight the necessity of carefully considering study design when analyzing markers that predict aggressive tumor biology, such as the *KRAS*-variant. Erroneous conclusions will otherwise be reached when using prevalence as a measure of the association with cancer risk if the populations studied have long ascertainment times. Such disparities in these and other areas of study cohort and design likely explain the failure to find the association between the *KRAS*-variant and sporadic ovarian cancer risk in a prior publication [13]. However, it is also important to highlight that the association found in this study is again strongest in women with a personal and family history most consistent with HBOC.

The finding that the *KRAS*-variant is associated with uninformative women with double primary cancer is important, as it further confirms that 1) the *KRAS*-variant is associated with uninformative HBOC families, 2) appropriate intervention for patients with the *KRAS-*variant who develop breast cancer may allow prevention of future ovarian cancer and 3) women with cancer that have the *KRAS-*variant may benefit from screening to detect additional cancer development at its earliest stages. Overall, this work continues to support the importance of the *KRAS*-variant broadly in cancer biology, and specifically in women’s health.
